# Impact of low triiodothyronine syndrome on long-term outcomes in patients with myocardial infarction with nonobstructive coronary arteries

**DOI:** 10.1080/07853890.2021.1931428

**Published:** 2021-05-26

**Authors:** Side Gao, Wenjian Ma, Sizhuang Huang, Xuze Lin, Mengyue Yu

**Affiliations:** Department of Cardiology, Fuwai Hospital, National Center for Cardiovascular Diseases, Chinese Academy of Medical Sciences and Peking Union Medical College, Beijing, China

**Keywords:** Myocardial infarction with nonobstructive coronary arteries (MINOCA), low T3 syndrome, cardiovascular outcomes

## Abstract

**Background:**

Low triiodothyronine syndrome (LT3S), frequently seen in patients with acute myocardial infarction (AMI), has been regarded as a predictor of poor outcomes after AMI. However, little is known about the prognostic value of LT3S in euthyroid patients with myocardial infarction with nonobstructive coronary arteries (MINOCA).

**Methods:**

A total of 1162 MINOCA patients were enrolled and divided into LT3S and no-LT3S groups. LT3S was defined as decreased free T3 (fT3 < 2.36 pg/mL) with normal values of thyroid-stimulating hormone. The primary endpoint was a composite of major adverse cardiovascular events (MACE), including all-cause death, nonfatal MI, stroke, revascularization, and hospitalization for unstable angina or heart failure. Kaplan–Meier, Cox regression, propensity score matching (PSM), and receiver-operating characteristic analyses were performed.

**Results:**

Patients with LT3S (prevalence of 17.5%) had a significantly higher incidence of MACE (19.6% vs. 12.9%; *p* = .013) than patients without during the median follow-up of 41.7 months. LT3S was closely associated with an increased risk of MACE even after multivariable adjustment (HR 1.50, 95% CI: 1.03–2.18, *p* = .037). After PSM, 197 pairs of patients with or without LT3S were identified, and LT3S remained a robust risk factor of worse outcomes (HR 1.53, 95% CI: 1.02–2.65, *p* = .042). Moreover, LT3S had an area under the curve (AUC) of 0.60 for predicting MACE. When adding LT3S to the thrombolysis in myocardial infarction (TIMI) risk score, the combined model yielded a significant improvement in discrimination for MACE.

**Conclusions:**

LT3S was independently associated with poor outcomes after MINOCA. Routine assessment of LT3S may provide valuable prognostic information in this specific population.

## Introduction

Acute myocardial infarction (AMI) remains a major cause of morbidity and mortality of cardiovascular diseases (CVD) worldwide [1], and more recently, a distinct population with myocardial infarction with nonobstructive coronary arteries (MINOCA) has been increasingly recognized due to the widespread use of coronary angiography. Among all AMIs, patients with MINOCA account for 5–10% and they are younger and more often women compared to those with AMI and obstructive coronary artery disease [2–5]. Till now, several studies have found that the prognosis of MINOCA is not trivial given that these patients are still at considerable risks for long-term adverse cardiovascular (CV) events despite the secondary prevention treatments [6–9]. Therefore, it is necessary to find potential residual risk factors and improve the prognosis in MINOCA patients.

Low triiodothyronine syndrome (LT3S), also known as non-thyroid illness syndrome (NTIS) or sick euthyroid syndrome, is characterized by decreased free triiodothyronine (fT3) without increased thyroid-stimulating hormone (TSH) levels during acute illness. In severely ill patients, free thyroxine (fT4) levels drop as well [10,11]. LT3S is commonly seen in AMI patients with an estimated prevalence of 15–20% [12,13], in which serum fT3 rapidly decreases after the onset of AMI with maximal changes during 24 h and 36 h. Changes in the metabolism of thyroid hormones (TH) such as decreased activation and increased inactivation of TH and altered feedback at the hypothalamic-pituitary level are the major causes of LT3S [14]. Lower fT3 levels may be detrimental for CV systems due totheir important role in post-ischemic left ventricular (LV) remodelling, maintaining cardiac function and mitochondrial integrity [12,13]. Recently, LT3S has been proved as an independent risk factor in patients with CV diseases (CVD) and is closely associated with increased risks of short- and long-term adverse events after AMI [15–22]. However, no relevant study has focussed on the clinical significance of LT3S in MINOCA patients. Here, we investigated the impact of LT3S on long-term prognosis after MINOCA and explored whether LT3S could facilitate risk prediction in this population.

## Methods

### Study population

**Figure 1.**
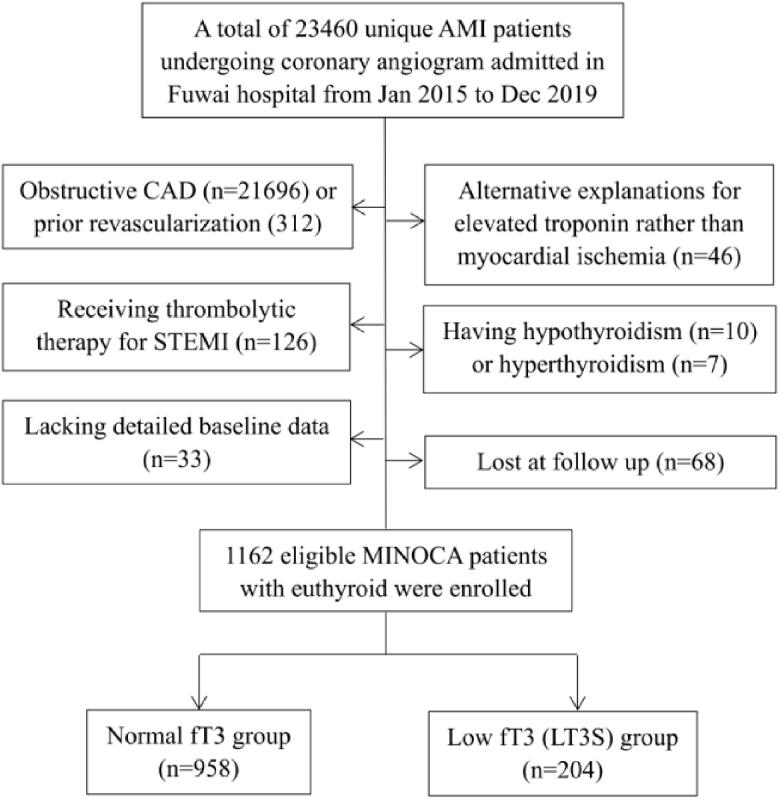
Study flowchart.

This was a single-centre, prospective and observational cohort study of patients with MINOCA. From January 2015 to December 2019, a total of 23,460 unique AMI patients with coronary angiograms were consecutively hospitalized in Fuwai hospital, including non-ST-segment elevation myocardial infarction (NSTEMI) and ST-segment elevation myocardial infarction (STEMI). Patients were diagnosed with MINOCA if they met the 4th universal definition of AMI [23] and the coronary angiography did not show stenosis of ≥50% in epicardial coronary arteries [2]. Patients were excluded due to: (1) presence of obstructive CAD (*n* = 21,696); (2) prior revascularization (*n* = 312); (3) thrombolytic therapy for STEMI since the coronary lesion may be affected by thrombolysis (*n* = 126); (4) alternate explanations for elevated troponin rather than coronary-related causes (e.g. acute heart failure, myocarditis, pulmonary embolism, Takotsubo syndrome, *n* = 46); (5) concomitant with hyperthyroidism (*n* = 7) or hypothyroidism (*n* = 10); (6) lack of detailed baseline data (*n* = 33); (7) lost at follow up (*n* = 68). As a result, 1162 eligible MINOCA patients with euthyroid were enrolled in the final analysis (Figure 1). Patients were prescribed the evidence-based secondary prevention treatment, including dual anti-platelet therapy (DAPT), statins, β-blocker, and angiotensin-converting enzyme inhibitor (ACEI) or angiotensin receptor antagonist (ARB) [24,25]. This study was approved by the Ethics Committee of Fuwai Hospital and complied with the Declaration of Helsinki. All enrolled subjects provided the written informed consent.

### Data collection

Patients’ demographics, medical history, laboratory tests, echocardiographic data and medication were collected and verified from in-person interviews and medical records. Body mass index (BMI) was calculated as weight(kg) divided by height (m) squared. Blood samples were routinely collected from cubital vein under fasting conditions for measuring thyroid and biochemical parameters. The thyroid function profiles including fT3, fT4, and TSH were measured using a direct chemiluminescence method (ADVIA Centaur, Siemens, USA). The reference intervals were set as follows: fT3, 2.36–4.21 pg/mL; fT4, 0.89–1.76 ng/dL; TSH, 0.55–4.78 μIU/mL. Serum concentrations of fasting blood glucose (FBG), low-density lipoprotein cholesterol (LDL-C), creatinine, and high-sensitive C-reactive protein (hs-CRP) were tested by an automatic biochemistry analyzer. The N-terminal po-B-type natriuretic peptide (NT-proBNP) at admission and peak cardiac troponin I (TnI) values were recorded. The left ventricular ejection fraction (LVEF) was measured by echocardiography using the biplane Simpson method. The Thrombolysis in Myocardial Infarction (TIMI) score was calculated since admission as previously described [26,27].

### Definitions and outcomes

In this study, euthyroidism was defined as having no history of hypothyroidism or hyperthyroidism and with a normal TSH level [13]. LT3S in euthyroid patients was defined as decreased serum fT3 level (fT3 < 2.36 pg/mL) with normal TSH during critical illness [10,11]. Diabetes mellitus (DM) was defined with FBG ≥7.0 mmol/L, 2-h plasma glucose ≥11.1 mmol/L, or having a history of DM. Hypertension was defined as repeated blood pressure ≥140/90 mmHg, past history, or taking anti-hypertensive drugs. Dyslipidemia was diagnosed by medical history or receiving lipid-lowering medications.

The primary study endpoint was a composite of major adverse cardiovascular events (MACE), including all-cause death, nonfatal MI, revascularization, nonfatal stroke, and hospitalization for unstable angina (UA) or heart failure (HF). The MACE was assessed as time to the first event. The secondary endpoints included each component of MACE and the composite “hard” endpoint of death, nonfatal MI, revascularization, and nonfatal stroke. Reinfarction was diagnosed according to the 4th universal definition of MI [23]. Revascularization was performed at the operator’s discretion due to recurrent ischaemia and the progression of the coronary lesion. Stroke was defined by the presence of neurological dysfunction and vascular brain injury caused by cerebral ischemia or hemorrhage [28]. Hospitalization for UA or HF reflected the clinical status and quality of life after AMI. Specifically, UA was diagnosed if the symptoms worsened with an increase in severity or length of anginal attacks [24]. HF was defined with the typical symptoms and evidence of a structural or functional cardiac abnormality [28]. Patients were regularly followed up at clinics or through telephone contact by a team of independent researchers. All the endpoints were confirmed by at least two professional cardiologists.

### Statistical analysis

Data were expressed as mean ± standard deviation (SD) or median with interquartile range for continuous variables and numbers with percentages for categorical variables. Differences were assessed using Student’s *t*-test or Mann–Whitney *U* test for continuous variables and Pearson’s *χ*^2^ or Fisher’s exact test for categorical variables. Cumulative incidence of MACE among groups was showed by Kaplan–Meier curve and compared using the log-rank test. The univariable and multivariable Cox proportional regression analyses were used to identify the association between LT3S and the outcomes. The risk of MACE was adjusted by age, sex, and MI classification (NSTEMI or STEMI) in Model 1 and further adjusted by multiple clinically relevant variables, including age, sex, MI type, hypertension, diabetes, dyslipidemia, LVEF, NT-proBNP, hs-CRP, and peak TnI. The hazard ratio (HR) with 95% confidence interval (CI) was calculated. To control the potential confounding effect of baseline characteristics differences, we performed a propensity score matching (PSM) analysis with a 1-to-1 match between LT3S and no-LT3S groups. Propensity scores were calculated by a binary logistic regression model. We assumed that the uneven distribution of baseline risk profiles was mainly due to the differences of age, sex, and MI classification, and thus these 3 factors were enrolled in the PSM model. Finally, 197 LT3S individuals were matched to 197 no-LT3S controls. The incidence and adjusted risk of MACE were also calculated after PSM. Accuracy was defined with areas under the curve (AUC) using a receiver-operating characteristic curve (ROC) analysis. The AUC values were interpreted as small (0.56–0.63), moderate (0.64–0.70), or strong (≥0.71) [29] and compared by Delong’s test [30]. To assess whether LT3S had an incremental predictive value for MACE when added to TIMI risk score, net reclassification index (NRI) and integrated discrimination improvement (IDI) were calculated to evaluate the improvement in discrimination [31]. A two-sided analysis with a *p*-value <.05 was considered statistically significant. Data were analyzed using SPSS version 22.0 (SPSS Inc., Chicago, USA) and R language version 3.6.3 (Feather Spray).

## Results

### Baseline characteristics

Patients were divided into normal fT3 group and low fT3 (LT3S) group based on the cut-off value of 2.36 pg/mL as previously described. The incidence of LT3S was 17.5% in all MINOCA patients (Figure 1). As shown in Table 1, patients with LT3S were older and more often female. They had a higher percent of STEMI and a higher prevalence of hypertension and diabetes. As expected, they had lower fT3, lower fT4, and higher TSH. LT3S group also had a higher Killip class, lower LVEF, higher TIMI score, and higher values of hs-CRP, NT-proBNP, and peak TnI. There were no significant differences in BMI, dyslipidemia, prior MI, creatinine, and LDL-C levels. The in-hospital medications were also similar among groups. In this regard, patients with LT3S had more baseline CV risk profiles compared to those with normal fT3 levels.

**Table 1.** Baseline characteristics and clinical outcomes in MINOCA patients with or without LT3S.

Variable	Total (*n* = 1162)	Normal fT3 (*n* = 958)	LT3S (*n* = 204)	*p*-Value
Male, *n* (%)	856 (73.6)	742 (77.4)	114 (55.8)	<.001
Age, years	55.6 ± 11.8	54.5 ± 11.5	61.0 ± 11.6	<.001
BMI, kg/m^2^	25.4 ± 3.7	25.5 ± 3.7	25.0 ± 4.0	.080
STEMI, *n* (%)	456 (39.2)	368 (38.4)	88 (43.1)	.042
Past history, *n* (%)				
Hypertension	619 (53.2)	497 (51.8)	122 (59.8)	.039
Diabetes	183 (15.7)	139 (14.5)	44 (21.5)	.012
Dyslipidemia	675 (58.0)	555 (57.9)	120 (58.8)	.815
Previous MI	58 (5.0)	48 (5.0)	10 (4.9)	.948
Killip class ≥ 2, *n* (%)	84 (7.2)	61 (6.3)	23 (11.2)	.027
LVEF, %	60.5 ± 7.4	60.9 ± 7.2	58.6 ± 8.2	.001
TIMI risk score	3.4 ± 1.3	3.2 ± 1.1	4.1 ± 1.5	.024
Laboratory test				
fT3, pg/mL	2.82 ± 0.40	2.95 ± 0.21	2.20 ± 0.15	<.001
fT4, ng/dL	1.16 ± 0.18	1.18 ± 0.18	1.07 ± 0.18	<.001
TSH, μIU/mL	2.11 ± 1.48	2.04 ± 1.34	2.40 ± 1.97	.001
FBG, mmol/L	5.69 ± 1.68	5.64 ± 1.58	6.00 ± 2.14	.007
LDL-C, mmol/L	2.29 ± 0.76	2.28 ± 0.76	2.33 ± 0.73	.368
Creatinine, μmol/L	80.3 ± 17.9	80.0 ± 16.8	82.4 ± 19.3	.176
hs-CRP, mg/L	2.2 (1.0, 5.6)	2.0 (0.9, 5.3)	4.3 (1.5, 9.6)	<.001
NT-proBNP, pg/mL	371 (112, 684)	363 (109, 677)	452 (138, 940)	.002
Peak TnI, ng/mL	3.4 (0.7, 6.5)	3.1 (0.5, 5.9)	5.6 (1.2, 8.7)	.006
In-hospital medication, *n* (%)				
DAPT	1078 (92.7)	891 (93.0)	187 (91.6)	.135
Statin	1113 (95.7)	919 (95.9)	194 (95.0)	.592
Beta-blocker	848 (72.9)	704 (73.4)	144 (70.5)	.397
ACEI or ARB	744 (64.0)	620 (64.7)	124 (60.7)	.288
CV outcomes, *n* (%)				
MACE	164 (14.1)	124 (12.9)	40 (19.6)	.013
Death, nonfatal MI, stroke or revascularization	99 (8.5)	70 (7.3)	29 (14.2)	<.001
All-cause death	16 (1.3)	9 (0.9)	7 (3.4)	.006
Nonfatal MI	40 (3.4)	28 (2.9)	12 (5.8)	.073
Revascularization	46 (3.9)	33 (3.4)	13 (6.3)	.092
Nonfatal stroke	12 (1.0)	8 (0.8)	4 (1.9)	.149
Hospitalization for UA	70 (6.0)	56 (5.8)	14 (6.8)	.376
Hospitalization for HF	46 (3.9)	31 (3.2)	15 (7.3)	.009

Low triiodothyronine syndrome (LT3S) was defined as decreased fT3 (fT3 < 2.36 pg/mL) with normal TSH levels.

BMI: body mass index; STEMI: ST-segment elevation myocardial infarction; LVEF: left ventricular ejection fraction; TIMI: Thrombolysis in Myocardial Infarction; fT3: free triiodothyronine; fT4: free thyroxine; TSH: thyroid-stimulating hormone; FBG: fasting blood glucose; LDL-C: low-density lipoprotein cholesterol; hs-CRP: high-sensitive C-reactive protein; NT-proBNP: N-terminal pro-B-type natriuretic peptide; TnI: Troponin I; DAPT: dual anti-platelet therapy; ACEI: angiotensin-converting enzyme inhibitor; ARB: angiotensin receptor antagonist; MACE: major adverse cardiovascular events; UA: unstable angina; HF: heart failure.

### Association between LT3S and outcomes

**Figure 2.**
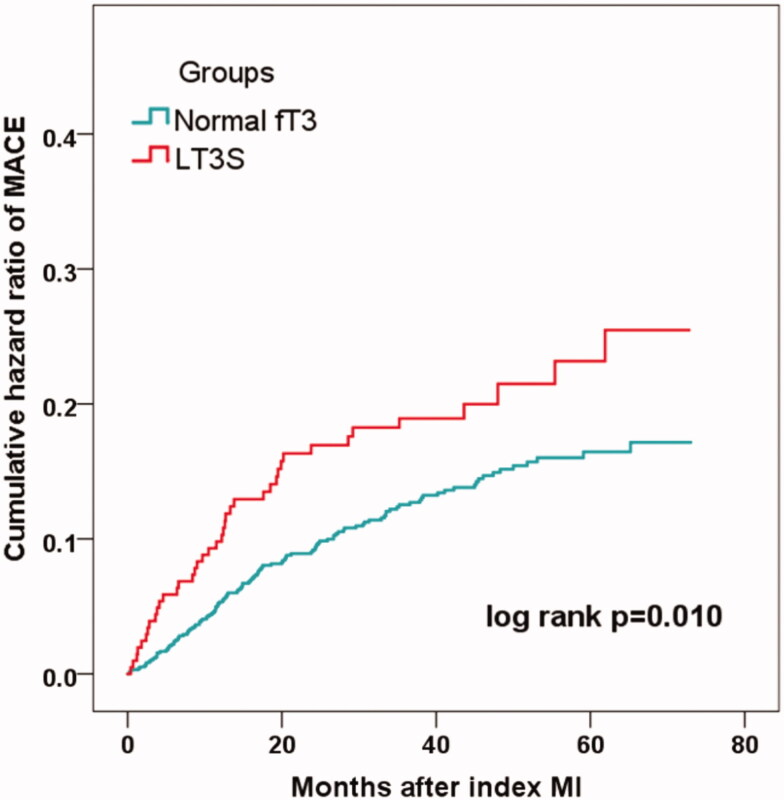
Incidence of MACE in MINOCA patients with or without LT3S.

Over the median follow-up of 41.7 months, 164 patients developed MACE (16 died, 40 had reinfarction, 46 had revascularization, 12 suffered stroke, 70 were hospitalized for UA and 46 hospitalized for HF) (Table 1). Patients with LT3S had a significantly higher incidence of MACE than patients without (19.6% vs. 12.9%; *p* = .013) (Table 1). The rate of the composite “hard” endpoint of death, recurrent MI, revascularization or stroke also markedly increased in the LT3S group (14.2% vs.7.3%; *p* < .001). In addition, the Kaplan–Meier analysis showed that the cumulative incidence of MACE was much higher in patients with LT3S (log-rank *p* = .010) (Figure 2).

At multivariate Cox analysis, the LT3S was significantly associated with an increased risk of MACE after adjustment for age, sex, and MI type (HR 1.66, 95% CI: 1.13–2.44, *p* = .011) or for multiple clinical variables (HR 1.50, 95% CI: 1.03–2.18, *p* = .037). The fT3 level was also correlated with the risk of MACE after multivariable adjustment (for per 1SD increase in fT3, HR 0.65, 95% CI: 0.44–0.98, *p* = .045) (Table 2). After PSM, 197 pairs of patients with or without LT3S were identified. The baseline demographic and comorbidities were comparable among the groups (Table 3). Even though, patients with LT3S still had a lower LVEF and higher levels of NT-proBNP, TnI and hs-CRP, indicating a more complicated AMI in the LT3S group. The incidence and adjusted risk of MACE also markedly increased in patients with LT3S (19.2% vs. 11.1%, *p* = .025; HR 1.53, 95% CI: 1.02–2.65, *p* = .042) after PSM, suggesting that LT3S was an independent risk factor of long-term MACE in MINOCA patients.

**Table 2.** Association between LT3S and the risk of MACE.

Group	Unadjusted		Model 1		Model 2	
	HR (95% CI)	*p*-Value	HR (95% CI)	*p*-Value	HR (95% CI)	*p*-Value
fT3, per 1SD increase	0.58 (0.40–0.85)	.006	0.62 (0.41–0.93)	.032	0.65 (0.44–0.98)	.045
Normal fT3	Reference		Reference		Reference	
LT3S	1.79 (1.25–2.57)	.001	1.66 (1.13–2.44)	.011	1.50 (1.03–2.18)	.037

Model 1 included age, sex, and MI type (NSTEM or STEMI). Model 2 included age, sex, MI type, hypertension, diabetes, dyslipidemia, LVEF level, NT-proBNP, peak TnI and hs-CRP in multivariate Cox analysis.

HR: hazard ratio; CI: confidence interval; SD: standard deviation; LT3S: low triiodothyronine syndrome.

**Table 3.** Distribution of clinically relevant variables and outcomes before and after propensity score matching in patients with or without LT3S.

Variables	Pre-PSM			Post-PSM		
	Normal fT3 (*n* = 958)	LT3S (*n* = 204)	*p*-Value	Normal fT3 (*n* = 197)	LT3S (*n* = 197)	*p*-Value
Baseline data						
Male, *n* (%)	742 (77.4)	114 (55.8)	<.001	97 (49.2)	98 (49.7)	.976
Age, years	54.5 ± 11.5	61.0 ± 11.6	<.001	61.0 ± 10.6	61.0 ± 11.4	.989
STEMI, *n* (%)	368 (38.4)	88 (43.1)	.042	85 (43.1)	84 (42.6)	.845
Hypertension, *n* (%)	497 (51.8)	122 (59.8)	.039	116 (58.8)	119 (60.4)	.758
Diabetes, *n* (%)	139 (14.5)	44 (21.5)	.012	33 (16.7)	43 (21.8)	.202
LVEF, %	60.9 ± 7.2	58.6 ± 8.2	.001	60.3 ± 5.9	58.7 ± 8.3	.002
hs-CRP, mg/L	2.0 (0.9, 5.3)	4.3 (1.5, 9.6)	<.001	1.9 (0.8, 4.6)	4.0 (1.4, 9.4)	<.001
NT-proBNP, pg/mL	363 (109, 677)	452 (138, 940)	.002	382 (104, 675)	447 (135, 936)	.013
Peak TnI, ng/mL	3.1 (0.5, 5.9)	5.6 (1.2, 8.7)	.006	3.7 (0.8, 6.4)	5.8 (1.1, 8.9)	.025
CV outcomes						
MACE	124 (12.9)	40 (19.6)	.013	22 (11.1)	38 (19.2)	.025
Risk of MACE	1 (reference)	1.50 (1.03–2.18)	.037	1 (reference)	1.53 (1.02–2.65)	.042

Clinically relevant variables and outcomes were compared before and after propensity score matching (PSM). Demographics and baseline comorbidities became comparable after PSM. The risk of MACE was adjusted by age, sex, MI type (NSTEM or STEMI), hypertension, diabetes, dyslipidemia, LVEF, NT-proBNP, peak TnI and hs-CRP, and expressed as HR (95% CI).

STEMI: ST-segment elevation myocardial infarction; LVEF: left ventricular ejection fraction; hs-CRP: high-sensitive C-reactive protein; NT-proBNP: N-terminal pro-B-type natriuretic peptide; TnI: Troponin I; MACE: major adverse cardiovascular events; LT3S: low triiodothyronine syndrome.

### Predictive value of LT3S for MACE

**Figure 3.**
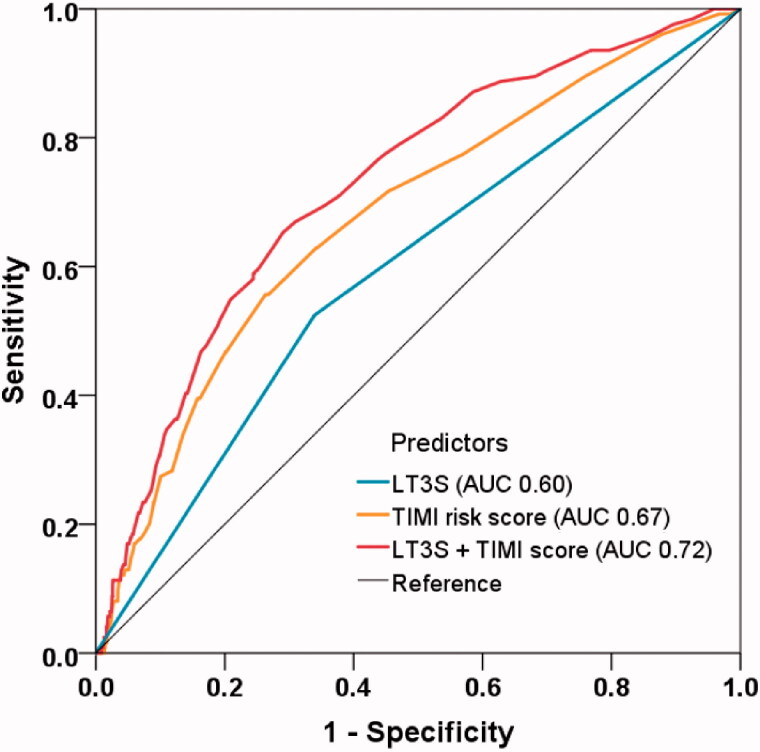
Model improvement in predicting MACE. Receiver operating characteristic curves showing the predictive value of LT3S, TIMI risk score, and the combined model incorporating LT3S and TIMI risk score using Cox regression. LT3S: low triiodothyronine syndrome; TIMI: thrombolysis in myocardial infarction; AUC: area under the curve.

The ROC analysis confirmed the ability of LT3S for MACE prediction (AUC 0.60, 95% CI: 0.54–0.65, *p* = .001), while the TIMI risk score had moderate discrimination for MACE (AUC 0.67, 95% CI: 0.62–0.72, *p* < .001) (Figure 3). When adding LT3S to the original TIMI score using Cox regression, the combined model enabled a more accurate prediction of MACE (AUC 0.72, 95% CI: 0.68–0.77, *p* < .001) and accordingly yielded a significant model improvement (ΔAUC 0.05, *p* = .021 by Delong’s test; NRI 0.536, 95% CI: 0.314–0.725, *p* = .001; IDI 0.044,95% CI: 0.012–0.075, *p* = .006) (Table 4).

**Table 4.** Model improvement for the TIMI score in combination with LT3S.

Models	AUC (95% CI)	*p*-Value	NRI (95% CI)	*p*-Value	IDI (95% CI)	*p*-Value
TIMI	0.67 (0.62–0.72)		Reference		Reference	
TIMI + LT3S	0.72 (0.68–0.77)	.021	0.536 (0.314–0.725)	.001	0.044 (0.012–0.075)	.006

TIMI: Thrombolysis in myocardial infarction; LT3S: low triiodothyronine syndrome; AUC: area under the curve; CI: confidence interval; NRI: net reclassification index; IDI: integrated discrimination improvement.

## Discussion

The present study, for the first time, verified the prognostic value of LT3S inpatients with MINOCA, and found that LT3S was independently associated with poor outcomes after MINOCA. Further, LT3S improved the accuracy of MACE prediction when added to the established TIMI risk score. These data support the routine assessment of LT3S for risk stratification in the contemporary real-world management of MINOCA.

MINOCA is a distinct clinical entity and represents a heterogeneous diagnosis with multiple underlying pathophysiological mechanisms, including plaque rupture, erosion, thromboembolism, coronary spasm, spontaneous dissection, microvascular dysfunction and supply/demand mismatch. Some non-ischaemic diseases such as myocarditis may also mimic the presentation of MINOCA [5]. More recently, the term MINOCA has been used to primarily describe patients with coronary-related ischaemia. We used these criteria and established a long-term cohort of MINOCA with a relatively large sample size. The prevalence of MINOCA is estimated to be 6% in all AMIs in a systematic review [4], which is close to the prevalence of 5.1% in our study. As reported, nearly one-third of MINOCA would present with STEMI, and MINOCA patients were more likely to be younger, female, and had fewer comorbidities [4]. We described the clinical profiles of MINOCA as well. Also, we found that the course of MINOCA was not benign. During the median follow-up of 3.5 years, 1.3% of MINOCA patients died and 14.1% of them developed MACE. Similarly, previous studies showed that MINOCA patients remained at high risk for long-term mortality and CV events [4–9]. The presence of traditional and novel risk factors and the lower use of long-term secondary prevention treatments may be responsible for the recurrent events after MINOCA [3,4]. Therefore, it is important to find risk factors contributing to this residual CV risk and further improve healthcare for this population.

LT3S in critically ill patients has been studied for decades, and recently, it has drawn more attention because of the close interplay between LT3S and CVD in acute settings. A meta-analysis revealed the pooled prevalence of LT3S in patients with CVD (21.7%), of which the highest prevalence was in heart failure (24.5%), followed by AMI (17.1%) [32]. Similarly, we found that 17.5% of patients had LT3S, indicating a remarkably high prevalence of LT3S in the MINOCA population. As reported, LT3S is a strong predictor of all-cause mortality, cardiac death and MACE in CVD patients, independently of known risk factors [15,32]. Recent studies further confirmed the clinical significance of LT3S in AMI patients, suggesting that lower circulating levels of fT3 are associated with a more complicated AMI including more severe myocardial injury, worse LV mechanics, and late recovery of cardiac function after 6 months [33–35]. LT3S also significantly correlated with poorer outcomes after AMI [15–22]. Of note, this is not only the case in the acute setting, but also in the longer term after recovery from AMI. In line with previous results, we found that patients with lower fT3 levels had a poorer prognosis after MINOCA. LT3S remained a robust predictor of MACE after multivariable adjustment and PSM. Adding LT3S to a traditional risk score further improved risk prediction. Taken together, these data confirmed the LT3S as a residual risk factor for MINOCA patients. Still, a causal relationship between LT3S and outcomes is yet to be concluded given the observational study design, and future randomized controlled trials are needed to validate our findings.

The underlying mechanisms linking the LT3S and poor CV outcomes have not been fully elucidated, yet several explanations have been proposed. In general, T3 is critical for cardioprotection. Both T3 and T4 constitute the active forms of TH, but only T3 is the bioactive TH for cardiomyocytes [12]. T3 modulates heart rate, cardiac contractility, and other cardiac functions via nongenomic and genomic approaches [36,37]. T3 can also enhance endothelial function, relax vascular smooth muscle, and thus reduce vascular resistance [38]. LT3S at the time of AMI, however, may exert a deleterious influence on CV systems such as reduced cardiac contractility, delayed diastolic filling and increased vascular resistance [12–14]. Till now, changes in cardiac structure and functions have been observed in both experimental and clinical studies involving LT3S or hypothyroidism [34–37], in which the circulating levels of fT3 correlated with cardiac remodelling and with lower LVEF both at early and follow-up periods after AMI. Some studies revealed that patients with LT3S tended to have higher levels of cardiac troponins and inflammatory markers that indicated more extensive myocardial damages following AMI [20,35]. Our study also found that patients with LT3S had lower LVEF and higher values of TnI and hs-CRP. Moreover, oxidative stress may also be involved as antioxidants could prevent the acute reduction of serum T3 levels in AMI patients [39]. All these pathophysiological changes may eventually contribute to an increased risk of CV events. Given the critical role of T3 in maintaining CV function, a number of studies have explored the efficacy of T3 dosing. It is found that early T3 supplementation can enhance LV functions and prevent ischaemic cardiac remodelling in experimental models [40,41]. In clinical trials, the T3 replacement therapy may partly increase cardiac output and lower systemic vascular resistance, however, it does not change outcomes despite its mild effects on myocardial performance [42–44], and its potential impact on long-term outcome has not been proved yet. Future studies are warranted to address the therapeutic relevance of LT3S after AMI.

## Limitation

Some limitations should be mentioned. First, the percentage of women was relatively low in our cohort, possibly due to the large proportion of men in all AMIs treated in our centre and a lower rate for women to receive coronary angiography. Given the potential selection bias in single-centre studies, future nationwide registry cohorts of MINOCA are warranted to validate our findings. Second, we did not capture and record the exact mechanism for every MINOCA patient. The association between aetiology of MINOCA and outcomes should be further investigated. Third, despite multivariate adjustment and PSM analyses were performed, there might be other unmeasured confounders that may affect the prognosis. Further, the fT3 levels were only measured at baseline, and the follow-up levels of fT3 may also be clinically significant.

## Conclusion

LT3S was an independent predictor of MACE after MINOCA. In clinical practice, special attention should be given to MINOCA patients with LT3S, who remain at high risks for long-term CV events.

## Data Availability

The present study was approved by the Ethics Committee of Fuwai Hospital. Datasets used or analyzed during the current study are available from the corresponding author on reasonable request.
